# The experiences of ferric carboxymaltose desensitization and provocation^☆^

**DOI:** 10.1016/j.waojou.2024.101025

**Published:** 2025-01-17

**Authors:** Fatma Dindar Çelik, Kurtuluş Aksu, Özgür Akkale, Hatice Çelik Tuğlu, Melis Yağdıran, Onur Telli, Gürgün Tuğçe Vural Solak, Enes Çelik

**Affiliations:** a University of Health Sciences, Ankara Ataturk Sanatorium Training and Research Hospital, Department of Immunology and Allergy, Ankara, Türkiye; b University of Health Sciences, Ankara Ataturk Sanatorium Training and Research Hospital, Department of Pediatric Allergy and Immunology, Ankara, Türkiye

**Keywords:** Drug provacation test, Drug hypersensitivity reactions, Ferric carboxymaltose, Iron sensitivity, Rapid drug desensitization

## Abstract

**Background:**

To present the characteristics of drug hypersensitivity reactions (DHR) among iron preparations and to describe the outcomes of rapid drug desensitization (RDD) and drug provocation tests (DPT) of ferric carboxymaltose (FCM).

**Methods:**

The retrospective descriptive study comprised patients with hypersensitivy to iron supplements. Low-risk according to the index reaction with iron, 10 patients underwent a 4-step DPT with FCM; an 11-step RDD protocol was administered to 21 patients not classified as low-risk. RDD success was evaluated for each cycle separately, defining successful completion as the implementation of all steps in both RDD and DPT protocols without subsequent early and/or late reactions.

**Results:**

Among the 21 patients (mean age: 41.73 ± 10.98 years, all female) hypersensitive to iron underwent FCM RDD, 20 patients (95.2%) successfully completed FCM treatment with RDD. RDD failed in only 1 patient (4.8%). The total number of desensitization cycles was 29, of which 28 (96.5%) were successful. Urticaria was the most common breakthrough hypersensitivity reaction and observed in 7 (33.3%) patients. Ten patients received FCM with DPT. The iron replacement therapy for these 10 patients was successfully completed. Urticaria developed in 2 patients after the completion of DPT.

**Conclusions:**

RDD is a dependable procedure facilitating the efficient delivery and completion of FCM treatments in patients with iron hypersensitivity. Additionally, FCM can be applied with DPT in low-risk patients.

## Introduction

Anemia is a global health disease associated with poor health outcomes, incrased morbidity and mortality. Iron deficiency is a common cause of anemia especially in females of childbearing age, children, and individuals living in low- and middle-income countries.^1^ Treatment modalities of iron-deficiency anemia vary depending on the degree of anemia. Severe symptomatic and life-threatening anemia should be treated with red blood cell transfusion. Oral and parenteral iron supplementations are the main treatment modalities in mild and moderate anemia. Parenteral iron replacement therapies are preferred when oral iron preparations cannot be administered due to side effects and in patients with impaired intestinal iron absorption due to gastrointestinal system diseases or gastrointestinal surgery and also when iron replacement therapy needs to be administered quickly.^2^

Parenteral iron replacement therapies are generally considered safe; however, they can rarely cause life-threatening severe hypersensitivity reactions (HSR). Two main immunological responses have been identified for iron hypersensitivity. First, IgE-mediated HSRs involve specific IgE molecules targeting the dextran moiety of intravenous (IV) iron preparations. Second, complement activation-related pseudoallergy (CARPA) is a non-IgE mediated HSR that occurs due to the rapid activation of the complement system upon exposure to substances, including iron-containing compounds.^3^ Ferric carboxymaltose (FCM), ferric gluconate, iron isomaltose, low molecular weight iron dextran, iron sucrose, and ferumoxytol are IV iron preparations used in many countries for the treatment of iron deficiency (Table 1). FCM is an iron complex comprising a ferric hydroxide core stabilized by a carbohydrate shell. This design enables controlled delivery of iron to reticuloendothelial system cells and subsequent transfer to iron-binding proteins such as ferritin and transferrin, minimizing the risk of large amounts of ionic iron release into the serum.^4^ It is non-dextran-containing, resulting in a low immunogenic potential, which consequently reduces the risk of anaphylactic reactions.^5^ FCM also allows the administration of a large amount of supplemental iron in a short period facilitates the rapid completion of anemia treatment.^6^

**Table 1:** Intravenous iron preparations

Iron product	Ferric carboxymaltose (FCM)	Ferric derisomaltose (iron isomaltoside)	Ferric gluconate (FG)	Ferumoxytol	Iron dextran, low molecular weight (LMW ID)	Iron sucrose (IS)
Examples of trade (brand) name	InjectaferFerinject	MonoferricMonofer	Ferrlecit	FerahemeRienso	InfedDexironCosmofer	Venofer
Carbohydrate shell	Carboxymaltose(branched polysaccharide)	Isomaltoside (linearoligosaccharide)	Gluconate(monosaccharide)	Polyglucose sorbitolCarboxymethylEther	Dextran (branchedpolysaccharide)	Sucrose(disaccharide)
Concentration of elemental iron (mg/ml	50	100	12.5	30	50	20
Maximum singledose	1000 mg	20–200 mg/kgbody weight	62.5–125 mg	510 mg	20 mg/kgbody weight	200–500 mg

Drug provocation tests (DPT) can be utilized to confirm or exclude drug allergies in selected low-risk patients (detailed in the “criteria selection” section). After obtaining a detailed medical history and excluding patients with chronic diseases (infections, uncontrolled asthma, underlying cardiac, hepatic, and renal diseases) that could exacerbate intensity of drug hypersensitivity reactions (DHR), DPT may be applied.^7^ Performing skin tests before DPT have been recommended especially for preparations containing dextran; however with the initiation of discussions regarding mechanisms such as CARPA, recent studies have indicated an increasing trend towards the administration of DPT without prior skin testing, particularly for iron preparations that do not contain dextran.^8^, ^9^, ^10^ Given the rarity of hypersensitivity reactions, the ability of IV iron to activate complement is not the only factor that leads to hypersensitivity. Other known risk factors that play a role in the development of hypersensitivity include patient characteristics such as genetic traits, underlying atopic conditions, advanced age, rate of drug administration, autoimmune diseases, and mastocytosis.^10^ While acquired temporary risk factors are exemplified by infectious diseases, certain medications, including nonsteroidal anti-inflammatory drugs, β-adrenoceptor blockers and ACE inhibitors, may also exacerbate the hypersensitivity reaction to iron.^9^^,^^11^ In patients carrying these risk factors, drug desensitization procedures may be considered.

Iron replacement therapies can be successfully administered using desensitization protocols in patients experiencing immediate hypersensitivity reactions. The parenteral iron desensitization methods used in clinical practice are still non-specific, non-evidence-based, and non-standardized protocols.^12^ Rapid drug desensitization (RDD) protocol using the 3-bag, 12-step approach is a safe and effective protocol for iron dextran, iron sucrose and FCM, enabling patients experiencing DHR of any severity to continue their treatment.^13^, ^14^, ^15^ However, the prolonged duration of treatment is a disadvantage of this 12- step protocol. Case series and clinical studies have reported that fewer than 12-step iron RDD protocols shorten the desensitization duration and show no disadvantage in terms of tolerance.^12^^,^^16^^,^^17^ Iron dextran has a longer history of clinical use in iron replacement therapies compared to FCM. For this reason, reports on iron dextran via RDD are more abundant.^15^^,^^18^, ^19^, ^20^, ^21^

In this study, we aimed to present the characteristics of DHRs among iron preparations and to describe the results of RDD and DPT of FCM.

## Material and methods

### Study design and ethical issues

This retrospective descriptive study was carried out at the allergy clinic in a tertiary training and research hospital. The study was carried out according to the ethical standards stated in the Declaration of Helsinki and its amendments, and all patients were examined and included with respect to good clinical practice guidelines. This study was conducted retrospectively; therefore, consent to participate was not obtained.

### Study population and data collection

Study population consisted of the subjects with indication for the use of intravenous FCM who were referred to the allergy clinic between January 2017 and December 2023 with the suspicion of hypersensitivity reactions to iron preparations. Demographic and clinical information of the patients, drug provocation and drug desensitization information were recorded from the patient's files and hospital information system. Those with missing or incomplete data were not included in the study.

Subjects’ signs and symptoms that occurred during and after the use of iron supplementation and their physical examination findings at the time of referral were noted*.* The diagnoses and management of DHR and anaphylaxis was made according to the guidelines of the European Academy of Allergy and Clinical Immunology (EAACI) and the American Academy of Allergy, Asthma and Immunology (AAAAI).^22^

### Criteria selection

The definition of low risk was established based on the European Academy of Allergy and Clinical Immunology/European Network on Drug Allergy (EAACI/ENDA) position paper on drug provocation testing.^23^ Patients with a history of hypersensitivity reactions that clearly did not require treatment or emergency medical attention; patients present with a non-urticarial exanthema that begins more than 6 h after drug intake, covers less than 50% of the body surface, and occurs without mucosal involvement, blisters, lymphadenopathy, eosinophilia, or systemic involvement, were defined as low risk. DPT was administered to patients classified as low-risk for iron hypersensitivity.^23^ Patients with a history of widespread urticarial rash, angioedema, anaphylaxis, wheezing, hoarseness, shortness of breath, respiratory arrest, palpitations, dizziness, hypotension, collapse, or the need for adrenaline therapy after iron administration were considered high-risk and underwent desensitization without drug provocation testing.^8^^,^^23^

DHRs were classified as early type, occurring within 6 h of iron preparation administration, and otherwise late type.^24^ Successful RDD was defined as completion of all 11 steps of the protocol, even if there were early and/or late reactions at intermediate steps.^25^

### Drug Provocation Tests

A four-step drug provocation test was performed with 2 vials of Ferinject® (the brand name of FCM) involving a total of 1000 mg iron. Each 10 mL Ferinject® vial contains 500 mg iron in the form of FCM. In the initial step 0.2 mL (10 mg) of the FCM solution was diluted with 5 mL of 0.9% sodium chloride and a total of 5.2 mL diluted solution administered intravenous push. At second step, 2.5 mL (125 mg) of FCM was diluted with 50 mL of 0.9% sodium chloride solution and a total of 52.5 mL of diluted solution administered as a 5-min infusion. Third, 7.3 mL (365 mg) of FCM was diluted with 100 mL of 0.9% sodium chloride solution and a total of 107.3 mL of diluted solution administered as a 10-min infusion. Finally, in the fourth step 10 mL (500 mg) of FCM was diluted with 100 mL of 0.9% sodium chloride solution and a total of 110 mL of diluted solution administered as a 15-min infusion (Table 2). The test proceeded to the next step if no reactions were observed, after 1 h observation time at per steps. After the completion of the DPT protocol, patients were clinically observed for 2 h.

**Table 2:** Ferric carboxymaltose Provocation Protocol

Dose number	FCM^a^ Dose		0.9% Sodium Chloride (mL)	Administered solution (mL)	Infusion Time^b^ (minutes)
	(mg)	(mL)			
1	10	0.2	5	5.2	Iv push
2	125	2.5	50	52.5	5
3	365	7.3	100	107.3	10
4	500	10	100	110	15

Iv: Intravenous.

a The brand name of the FCM medication used is Ferinject, and a total of 2 vials (each containing 500 mg/10 mL of iron) are administered.

b One-hour waiting period is maintained between each step of the DPT, and if hypersensitivity reactions are not observed, the procedure advances to the next step

Patients who experienced an observable signs and symptoms include skin rash, pruritus, urticaria, angioedema, nausea and vomiting, abdominal pain, rhinorrhea, dyspnea, tachycardia, hypotension, and anaphylaxis during the procedure were considered to have a positive DPT.^23^

### Desensitization protocol

The FCM desensitization protocol of Montandon et al^16^ was used with some modifications. Due to stability reasons, FCM should not be diluted to concentrations lower than 2 mg iron/mL. So a stock solution with a concentration of 2 mg iron/mL of FCM was prepared. This was achieved by adding a vial (10 mL) of 500 mg FCM to 250 mL 0.9% sodium chloride solution.^21^

The infusion was initiated by administering 0.25 mg of FCM over a 15-min period at a rate of 0.5 mL/h. Subsequent doses were administered at 15-min intervals with an increasing rate until a rate of 250 mL/h is reached. Afterwards, the next 500 mg of FCM in a 250 mL solution of 0.9% sodium chloride was infused over a 1-h period. At the end of this 4-h desensitization protocol, the patients received a total of 1000 mg of FCM (Table 3). After the completion of the RDD protocol, patients were clinically observed for 2 h.

**Table 3:** Ferric carboxymaltose desensitization protocol.

Dose number	Infusion Rate (ml/Hour)	Dose (mg)	Cumulative Dose (mg)	Infusion Time (minutes)
1	0.5	0.25	0.25	15
2	1	0.5	0.75	15
3	2	1	1.75	15
4	4	2	3.75	15
5	8	4	7.75	15
6	16	8	15.75	15
7	32	16	31.75	15
8	64	32	63.75	15
9	128	64	127.75	15
10	250	375	500	45
11	250	500	1000	60

The stock solution was prepared with 500 mg of ferric carboxymaltose added to 250 mL of 0.9% sodium chloride solution

Breakthrough hypersensitivity reactions (B-HR) was defined as a hypersensitivity reaction occurring during or after administration of FCM via RDD. When a B-HR occurs during the protocol, the infusion is stopped and BHR is managed through medical treatment (H1 and H2 blockers and/or methylprednisolone). Then the RDD reverts one step prior to the BHR. An additional intermediate dose step is introduced between the reverted step and the step that the B-HR occurred. The RDD protocol is restarted from the reverted step, continues with the newly added intermediate step and then with the subsequent steps. For instance, when B-HR occurs at the 10th step, the protocol reverts to the 9th step to restart with 128 mL/h infusion rate. A new infusion dose of 190 mL/h is introduced between the 9th and 10th steps. Then continue with 250 mL/h infusion.

### Statistical analysis

Descriptive statistics were obtained by using the IBM SPSS Statistics for Windows, Version 25.0 (IBM Corp., Armonk, NY, USA). Data are summarized as mean ± standard deviation (minimum - maximum) or median with interquartile range for continuous variables and as frequency (percentage) for categorical variables.

## Results

During the period covered by our study, a total of 30 patients underwent DPT and/or RDD with FCM, and their data were evaluated (Table 4). Hypersensitivity to iron preparations was diagnosed in 27 patients based on their history of reactions to iron agents. Skin tests with FCM were not performed prior to DPT or RDD. The remaining 3 patients had a history of hypersensitivity reactions to non-iron medications and underwent DPT with FCM due to their reluctance to use iron agents. 7 of the 27 patients were evaluated as low risk and DPT with FCM was performed on these patients. A total of 10 patients underwent DPT, all tolerated FCM without symptoms between the steps and successfully received their planned iron therapy. But 2 patients developed urticaria within 10 min after the conclusion of the DPT procedure. One of these patient, the iron administered during DPT was considered adequate and no additional iron replacement was required. In the other patient iron replacement was required again 3 months later. Due to the development of urticaria after the DPT procedure, this patient received FCM through RDD.

**Table 4:** Characteristics of cases who were received FCM desensitization or provacation (n = 30).

	
Age (years)	41.73 ± 10.98 (19–70)
Gender	
Male	0 (0%)
Female^a^	30 (100%)
Comorbitities	
Asthma	6 (20%)
Diabetes mellitus	1 (3.3%)
Hypertension	2 (6.7%)
Psychiatric disorder^b^	2 (6.7%)
Gastric ulcer	1 (3.3%)
Causes of anemia	
Menorrhagia	22 (73.3%)
Gastrointestinal disorders	7 (23.3%)
Low dietary iron intake	1 (3.3%)
Hemoglobin (g/dL)	9.4 ± 1.83 (4.7–12)
Ferritin (mcg/L, median ± IQR)	4.0 (3.3–6.8)
Previous HSR history^c^	
Ferrous iron (PO)	18 (60%)
Ferric iron (PO)	6 (20%)
Ferric carboxymaltose	8 (26.7%)
Ferric hydroxide sucrose	1 (3.3%)
The procedure performed	
Rapid drug desensitization	29 cycle (21 patient)^d^
Drug provocation test	10 cycle (10 patient)
Type of drug hypersensitivity reaction (n = 27)	
Early	24 (88.9%)
Late	3 (11.1%)
Symptoms^e^	
Skin rash	16 (53.3%)
Pruritus	20 (66.7%)
Urticeria	16 (53.3%)
Angioedema	3 (10%)
Nausea & vomiting	2 (6.7%)
Abdominal pain	2 (6.7%)
Rhinorrhea	1 (3.3%)
Dyspnea	3 (10%)
Palpitation	1 (3.3%)
Light-headednes	3 (10%)
Anaphylaxis	1 (3.3%)

Data are summarized as mean ± standard deviation (minimum-maximum) for continuous variables and as frequency (percentage) for categorical variables. HSR: Hypersensitivity reactions, IQR: interquartile range, PO: peroral

a None of the patients were pregnant.

b Both gastrointestinal iron loss and iron absorption disorders.

c In some cases, multiple iron allergies were observed.

d Both provocation and desensitization were performed in one patient.

e Symptoms that develop during the index reaction

FCM desensitization was performed in 21 patients hypersensitive to iron. The total number of desensitization cycles were 29. Anaphylaxis developed in 2 cycles. In one patient anaphylaxis occurred at step 10 of RDD, manifesting with dyspnea, hypotension, and nausea. Intramuscular adrenaline was administered and when the symptoms regressed, the procedure resumed from step 9. Intramuscular pheniramine hydrogen maleate, intravenous methylprednisolone, and oral montelukast were administered as medication prior to the this step. Afterwards, the protocol was continued and completed successfully. But in the other patient, anaphylaxis occurred at stage 10 (urticaria, abdominal pain, hypotension) desensitization was discontinued due to the prolonged duration required to control the patient's symptoms with medical treatment and RDD was terminated. The characteristics of the 2 patients who experienced anaphylaxis during RDD are given in Table 5.

**Table 5:** Characteristics of patients with anaphylaxis during rapid drug desensitization.

	Patient 1	Patient 2
Age	36	28
Sex	Female	Female
Causes of anemia	Menorrhagia	Atrophic gastritis
Hemoglobin (g/dL)	8	9.3
Ferritin (mcg/L)	3	3.3
Comorbidities		
Asthma	No	No
Gastric diseases	No	Yes
Atopy	No	No
Previous history of drug hypersensitivity other than iron	No	No
Previous history iron	FCM	Iron (II)-glycine sulfate (P.O)
İndex hypersensitivity reaction	Urticaria	Skin rash
Type of hypersensitivity reaction	Early	Early
RDD completed with premedication	Yes	No
RDD: Rapid drug desensitization		

Desensitization was completed in 28 (96.6%) cycles and could not be completed in 1 (3.4%) cycle. Urticaria developed as B-HR in a total of 7 cycles (24.1%), occurring in 2 cycles between the steps and in 5 cycles within the first hour after RDD protocol was completed. Pheniramine hydrogen maleate and methylprednisolone were administered to patients who developed urticaria for symptom control.^26^ Two cycles in which urticaria developed between the steps were completed RDD with an intermediate step added after controlling urticaria with medication. 5 patients who developed urticaria within 1 h after the completion of RDD were observed for 2 h after symptom control was achieved. Only 1 patient required an FCM RDD again. For this patient, an infusion dose of 190 mL/h was added between the 9th and 10th steps and the RDD was completed without any B-HR. There were no deaths due to desensitization procedures in our series.

## Discussion

Intravenous ferric carboxymaltose provides rapid and high-dose iron replacement when oral iron supplements are not tolerated or in the presence of gastrointestinal malabsorption.^27^ It is the preferred option for treatment; however, there is still a very low risk of serious immediate HSR.^28^ Hesitation in intravenous iron administration due to patients' previous history of iron hypersensitivity may cause delays in anemia treatment. RDD has allowed anemia patients to receive their treatments on time.

Iron deficiency anemia is a common disorder worldwide, occurring more frequently in women than in men.^29^ The need for iron treatment and the incidence of hypersensitivity reactions to iron preparations are higher in women.^30^^,^^31^ Similarly, in the present report, the entire study population consisted of female patients.

FCM application via RDD was proven to be effective in previous case reports and case series. In a case series by Di Girolamo et al,^19^ RDD was performed in 3 patients; 2 with oral sodium ferric gluconate, the culprit agent, and 1 with FCM due to a history of DHR to an oral iron preparation containing ferric pyrophosphate. They reported that all procedures were successful, and none of the patients experienced any reactions^.^ In a 10-year clinical observational study conducted by Varandas et al,^14^ a total of 9 cycles of RDD with culprit agents (7 iron sucrose, 2 FCM) were administered to 3 patients. The study reported 2 successful cycles (1 iron sucrose and 1 FCM). However in the third case, despite premedication, 7 cycles of RDD could not be completed due to severe hypersensitivity reactions. In both studies, Castell's 3-bag 12-step method was employed for FCM desensitization, and the extended duration of this protocol is its primary limitation.^13^

Montandon et al^16^ reported a case series of successful intravenous iron desensitization with FCM using single-bag dilution in 3 patients with previous anaphylaxis with iron formulations. The shorter duration of this protocol made it more convenient. A case of successful FCM desensitization was previously reported by modifying the Montandon protocol in a patient where the culprit agent was FCM.^21^ Later, Özden et al^12^ reported that they performed successful RDD with FCM in 15 patients with a history of sensitivity to iron preparations using the same protocol, and no reactions developed in the patients during the procedure.

Our study, in which RDD with FCM was applied to 21 patients, represents the study with the highest number of RDD cases with FCM in the literature. With the modified protocol of Montandon et al.,^16^ 96.6% (n = 28) of the administered cycles were successful. Anaphylaxis developed in 2 cycles (6.8%) of a total of 29 cycles. Urticaria was the most frequent B-HR symptom developed during RDD procedure.

Although routine use of premedication before RDD may be useful in preventing hypersensitivity reactions. But the agents used for premedication may also lead to adverse reactions and drug interactions.^32^ The role of H1 antihistamines in particular has recently been questioned, their benefits are unclear, and they do indeed sometimes increase tachycardia, hypotension, and somnolence.^33^ Therefore, routine premedication before RDD is not recommended.^11^^,^^34^^,^^35^ In the case series, it is seen that premedication is generally applied in desensitization procedures with iron dextran, which has a higher allergenic potential.^15^^,^^20^^,^^22^^,^^36^ In our study, no patient received routine premedication before RDD. Premedication was administered only after B-HR occurred in order to complete RDD procedure.

In low-risk patients with iron hypersensitivity, DPT with FCM can be performed. DPT is the only definitive way to determine whether a patient can tolerate a medication and the gold standard for drug allergy testing. In this study, FCM provocation was conducted in 10 patients. Urticaria developed within the 10 min after procedure completed in 2 patients. The FCM provocation was completed in 8 patients without any HSR. DPT carries a small risk of serious reactions even when administered to low-risk patients. The patients should be selected properly, provocation and desensitization procedures should be performed by experienced clinics.^8^

Patient's risk stratification is conducted based on factors such as the severity and suspected mechanism of the index reaction, the implicated drugs and the clinical characteristics of the patient.^10^ It may be useful to perform skin tests (skin prick test, intradermal test, patch test) before applying DPT. But skin tests is not always necessary in low-risk patients.^23^ In this study, no skin tests were performed on the patients before DPT application and no serious immediate hypersensitivity reaction was observed in any patient. This shows that the risk analysis was carried out correctly in the cases. In the study by Stojanovic et al.^37^ a rechallenge infusion (generally at standard infusion rates) with FCM was performed on 60 patients with a history of HSR to non-FCM intravenous iron preparations and the infusion could not be completed in 1 patient due to an immediate reaction characterized by chest tightness and nausea.^31^ RDD can provide the completion of FCM treatment in patients who experience HSR during standard infusion rates.

The main limitation of this study is its retrospective design, which limits the exploration of additional data. Another limitation of the study is that DPT or RDD was not to the index drug in some cases. Furthermore, patients who tolerated RDD were not re-challenged with DPT or tryptase or other biochemical mediators were not obtained at the time of reactions to help endotype the reactions.

The present study investigates FCM administration via RDD in iron hypersensitive patients. RDD is a reliable procedure that facilitates the safe and effective delivery of FCM treatments in these patients. Additionally, data on FCM administration via DPT are included in this study, contributing further to the literature. DPT application may be a viable option for low-risk patients.

## Abbreviations

**CARPA**, Complement Activation-Related Pseudoallergy; **B-HR**, Breakthrough Hypersensitivity Reactions; **DHR**, Drug Hypersensitivity Reactions; **DPT**, Drug Provocation Tests; **EAACI**, European Academy of Allergy and Clinical Immunology; **ENDA**, European Network on Drug Allergy; **FCM**, Ferric Carboxymaltose; **HSR**, Hypersensitivity Reactions; **RDD**, Rapid Drug Desensitization.

## Funding

None.

## Ethical approval

Ethical approval for the study was obtained from the Clinical Research Ethics Committee of Ataturk Sanatorium Training and Research Hospital in Ankara, Turkey (February 14, 2024/2024-BÇEK/16). The study was carried out according to the ethical standards stated in the Declaration of Helsinki and its amendments, and all patients were examined and included with respect to good clinical practice guidelines. This study was conducted retrospectively; therefore, consent to participate was not obtained.

## Author contributions

Kurtuluş Aksu constructed the research hypothesis; all authors contributed substantially to the study design; Fatma Dindar Çelik, Kurtuluş Aksu, Enes Çelik, Özgür Akkale, Hatice Çelik Tuğlu, Melis Yağdıran, Onur Telli, Gürgün Tuğçe Vural Solak contributed substantially to data collection; Fatma Dindar Çelik, Kurtuluş Aksu and Enes Çelik performed data analysis and interpretation; Fatma Dindar Çelik, Kurtuluş Aksu and Enes Çelik substantially contributed to the writing of the manuscript; all authors reviewed the final manuscript, contributed to essential revisions, and approved the manuscript.

## Authors' consent for publication

All authors have consented to the publication of the manuscript.

## Data availability

The data set used and/or analyzed during the present study is available upon reasonable request.

## Declaration of competing interest

All authors declare to disclose no conflict of interest may have influenced either the conduct or the presentation of the research.
